# Impact of adversity on early childhood growth & development in rural India: Findings from the early life stress sub-study of the SPRING cluster randomised controlled trial (SPRING-ELS)

**DOI:** 10.1371/journal.pone.0209122

**Published:** 2019-01-09

**Authors:** Sunil Bhopal, Reetabrata Roy, Deepali Verma, Divya Kumar, Bilal Avan, Bushra Khan, Lu Gram, Kamalkant Sharma, Seeba Amenga-Etego, Satya Narayan Panchal, Seyi Soremekun, Gauri Divan, Betty R. Kirkwood

**Affiliations:** 1 Maternal & Child Health Intervention Research Group, Department of Population Health, Faculty of Epidemiology & Population Health, London School of Hygiene & Tropical Medicine, London, United Kingdom; 2 Northern School of Paediatrics, Newcastle upon Tyne, United Kingdom; 3 Sangath, New Delhi, India; 4 Department of Psychology, University of Karachi, Karachi, Pakistan; 5 Institute for Global Health, University College London, London, United Kingdom; 6 Kintampo Health Research Centre, Brong Ahafo, Ghana; Harvard TH Chan School of Public Health, UNITED STATES

## Abstract

**Introduction:**

Early childhood development is key to achieving the Sustainable Development Goals and can be negatively influenced by many different adversities including violence in the home, neglect, abuse and parental ill-health. We set out to quantify the extent to which multiple adversities are associated with impaired early childhood growth & development.

**Methods:**

This was a substudy of the SPRING cluster randomised controlled trial covering the whole population of 120 villages of rural India. We assessed all children born from 18 June 2015 for adversities in the first year of life and summed these to make a total cumulative adversity score, and four subscale scores. We assessed the association of each of these with weight-for-age z-score, length-for-age z-score, and the motor, cognitive and language developmental scales of the Bayley Scales of Infant Development III assessed at 18 months.

**Results:**

We enrolled 1726 children soon after birth and assessed 1273 of these at both 12 and 18 months of age. There were consistent and strongly negative relationships between all measures of childhood adversity and all five child growth & development outcome measures at 18 months of age. For the Bayley motor scale, each additional adversity was associated with a 1.1 point decrease (95%CI -1.3, -0.9); for the cognitive scales this was 0.8 points (95%CI -1.0, -0.6); and for language this was 1.4 points (95%CI -1.9, -1.1). Similarly for growth, each additional adversity was associated with a -0.09 change in weight-for-age z-score (-0.11, -0.06) and -0.12 change in height-for-age z-score (-0.14, -0.09).

**Discussion:**

Our results are the first from a large population-based study in a low/middle-income country to show that each increase in adversity in multiple domains increases risk to child growth and development at a very early age. There is an urgent need to act to improve these outcomes for young children in LMICs and these findings suggest that Early Childhood programmes should prioritise early childhood adversity because of its impact on developmental inequities from the very start.

## Introduction

Childhood development is key to achieving the ambitious global Sustainable Development Goals[1,2], particularly aspects of goals 1 (poverty reduction), 2 (nutrition), 3 (good health & wellbeing), 4 (school readiness), and 16 (violence reduction). Improving the development of young children will lead to improved health and wellbeing across the lifecourse, indeed children who were part of the early childhood home-visiting trials in Jamaica have now been followed into adulthood where the effects of this early years intervention are sustained in terms of increased employment & earnings[3]. The global child health community has placed early childhood development (ECD) high on the health agenda, and the Lancet Series *Advancing Early Childhood Development*: *from Science to Scale* served as a call to action, arguing that getting children off to a good start in life will reap benefits in health and wellbeing for “today's children, tomorrow's adults, and for future generations”[1].

Optimal child development starts before conception and is dependent on adequate nutrition for mother & child, protection from threats, provision of learning opportunities, and caregiver-interactions that are stimulating, responsive, & emotionally supportive. This whole environment around a child is conceptualised in the World Health Organization’s Nurturing Care Framework for Early Childhood Development[4] which was presented at the World Health Assembly in May 2018. The focus is on the ‘first thousand days’—the period from conception through the first two years of a child’s life—because of the adaptability of children’s brains during this period and because reversing early deficits becomes more difficult as children grow older[5].

Optimal development in early childhood can be knocked off course by a whole range of factors concerning a child’s environments and relationships with caregivers. These ‘adversities’ vary in intensity and include for example, violence in the home, neglect, abuse, lack of opportunity for play & cognitive stimulation, and parental ill-health[6,7]. Whilst each of these has the potential to cause problems for a child growing up, exposure to multiple adversities simultaneously poses a cumulative burden and is even more detrimental to a child’s wellbeing. This is all the more important in low- and middle-income countries where children are exposed to multiple low-level risks [8,9] with attended negative consequences. Empirical evidence has been presented from LMICs including in Guatemala where Gorman & Pollitt describe a linear relationship between cumulative psychosocial risk and cognition [10], Sri Lanka where increasing number of traumatic events was associated with impaired development [11] as well as the described associations between cumulative risk and adjustment disorder and PTSD [12,13]. However, there is minimal evidence from the crucial period when children are 0–2 years old from LMICs. Given the broad range of adversities faced by children in LMICs and impacts of these on health and wellbeing, we set out to quantify the extent to which these negatively affect child growth & development in the very early years, and aimed to assess the relative contribution of different groups of adversities In this paper we analyse the role of childhood adversity through pregnancy & the early years on early childhood development amongst children enrolled in the Early Life Stress sub-study of the SPRING randomised controlled trial (SPRING-ELS) in Haryana state, India–the country with the largest number of young children at extreme risk of impaired cognitive and social-emotional development[14].

## Methods

### Overview of study design

SPRING-ELS was a sub-study of the Wellcome Trust funded SPRING cluster randomised controlled trial in India analysing stress and adversity in young children. Details on SPRING are presented elsewhere[15] but in brief SPRING in India developed an innovative, feasible, affordable & sustainable community-based approach to delivering a home visiting programme through a new cadre of community-based worker with the aim to improve early childhood growth and development. SPRING was designed from the outset to be feasible and scalable through the governmental health system. A parallel trial was done in Pakistan with the same aim but working through existing health system structures with an existing cadre of worker. SPRING was evaluated by parallel cluster randomised controlled trials with clusters in India defined as the catchment area of functioning health sub-centres, the lowest level of the Indian primary healthcare system. There were 24 clusters. Primary outcomes were height-for-age, the best early childhood predictor of human capital[16], and Bayley Scales of Infant Development III (BSID-III), the gold standard assessment of a child’s development in the early years[15]. These impact outcomes were complemented by in-depth economic analysis, process-evaluation and a broad range of intermediate outcomes selected based on a conceptual-framework. This additional work will inform unpacking of the SPRING causal pathway, provide deeper understanding of mechanisms of trial impact, and inform lessons for scale-up and incorporation into health systems. SPRING took place in 120 villages across three administrative blocks of Rewari district, Haryana state, India. The total population was around 200,000. Rewari district is predominantly rural and has health and demographic indicators around average for the state. The overall literacy rate in Haryana is 76%, with female literacy of 67%[17]. The sex ratio is 879 females per 1000 males[17]–amongst the lowest ratio in India. Infant mortality is 41/1000 live births[18]–around the national average. More than one third of under-five year old children are stunted[19]. The SPRING trial is registered with ClinicalTrials.gov, number NCT02059863.

### Data collection

Participating SPRING mothers and their children were identified by an ongoing trial surveillance system whereby trained resident fieldworkers visited every household in the study area every 8 weeks to identify pregnancies and births, and follow-up pregnant women & children already identified. Surveillance system fieldworkers collected the demographic & socioeconomic data used in this study on custom-programmed mobile phones at enrolment. A separate group of fieldworkers did detailed SPRING assessments with children and their mothers when children turned 12 & 18 months of age. Adversity assessments were done at 12 months with mothers and children where the child was planned to have outcome assessments at 18 months of age. This was at least the first 50 children born in each of the 24 clusters after the trial start date (18 June 2015). These detailed assessments were spread out over two days in order to reduce the burden to participants and took a total of around 2.5 hours. All questionnaires were asked of the mother, and observations were done with both the mother and child. Other assessments during this two day visit that are not described in this paper include anthropometry, a feeding questionnaire, a maternal knowledge questionnaire and saliva and hair sampling for stress biomarker analysis.

#### Childhood adversity measures

To develop the set of adversities, we carried out formative research with local mothers and grandmothers, took advice from child development experts and reviewed the literature on existing evidence and tools. We selected 22 adversities with a focus on those adversities operating at the household level and did not consider those operating more widely because young children in this setting spend most of their time and interact most closely with family members inside the home and these adversities are therefore of most importance to these young children. Three of the adversities were assessed at enrolment, and the other 19 were assessed at 12 month assessment (Table 1).

**Table 1:** Childhood adversities in SPRING-ELS sub-study. prevalence and proportion of imputated values.

Domain	Items	Prevalence	Imputation
Socioeconomic	1. Socioeconomic status: lowest quintile of asset index (*E) ^a^	20.0%	
	2. Father education level: primary or none(*E)	5.0%	
	3. Mother education: none or 1–5 grades (*E)	11.9%	
	4. Father occupation: at home, seasonably employed or casual labourer	24.7%	
	5. Mother married under legal age (18 years)	20.0%	41 (3.2%)
	6. Family debt ^b^ or mother reports being unable to afford food for self or child at any point ^c^	18.0%	
Maternal Stress	1. Mother reports death of husband, parent, sibling, child or friend since pregnancy	5.4%	
	2. Mother seriously injured or ill since pregnancy	4.0%	
	3. Any violence from husband or mistreated by any other person since pregnancy ^d^	13.4%	
	4. PHQ9 score > = 5 or problems described make it very/extremely difficult to do daily activities	19.5%	23 (1.8%)
	5. Duke social support & stress scale: support < = 40 or stress >27	6.3%	23 (1.8%)
	6. Husband’s alcohol use causes problems for mother ^e^	8.3%	
Relationship	1. Any of mother, father, mother or mother-in-law were “unhappy” when found out child was a girl ^f^	15.2%	
	2. Mother’s Object Relations Scale concern level: moderate or high	50.4%	
	3. Observed feeding style: very low quality ^g^	13.3%	418 (32.8%)
	4. HOME inventory^h^ score: lowest quintile	15.6%^i^	1 (0.07%)
Child	1. Mother-reported child born prematurely	10.2%	
	2. Child admitted to hospital any time during first year of life	14.9%	
	3. Mother & child separated for one week or more during first year of life	1.7%	
	4. Child left alone or with child under 10 years for more than one hour in the past week	4.6%	
	5. Older children who live in house: say anything to make child cry or unhappy (in last week)	30.5%	
	6.Older children who live in house: hit/punched/kicked/bit child on purpose to make them unhappy (in last week)	17.9%	

^a^ SES score calculated with principle components analysis using data on mother, household demographics and animal & asset ownership

^b^ Answered yes to question: “Since you became pregnant, have you or your immediate family who live with you been in debt?”

^c^ Answered yes to question: “Since you became pregnant, have you ever been hungry because you could not afford to buy food?” or similar related to child

^d^ Using WHO multi-country study on women’s health and domestic violence against women

^e^ If woman reported husband drinking alcohol, answered yes to question: “does this cause any problems for you”

^f^ Question: “When [person] found out your baby was a girl were you/they happy, unhappy or didn’t mind whether you had a girl or a boy?”

^g^ Assessed using observed feeding index. Very low quality means < = 1 positive verbalisations, and < = 1 games played and < = 1 responsive actions, plus > = 1 negative actions by mother towards child during feeding session

^h^ The Home Observation for the Measurement of the Environment Inventory

^i^ Not exactly 20% because cut-off made at HOME score of 27

*E All items were assessed at 12 months of age except those marked *E which were collected at enrolment into the surveillance system

**Fig 1:**
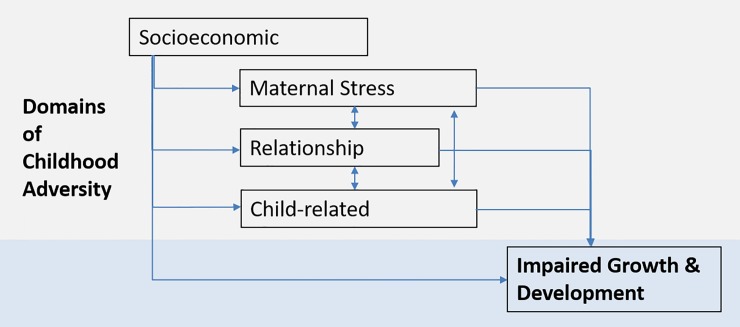
Conceptual framework linking domains of childhood adversity to suboptimal growth and development.

To explore further we examined the relative importance of particular groups of adversities based on a conceptual framework starting with direct adversities for a child with links to more distal adversities including maternal stress and difficulties in the carer-child relationships and the more overarching socio-economic factors (Fig 1).

For the second part of this analysis, the 22 adversities were therefore grouped as follows: 1) household-level socio-economic factors, 2) maternal stressors, 3) child-carer relationships and 4) child-related factors and are described within these groups below.

**Group 1—Socioeconomic—Consists of five factors:** 1) Asset index—being in the lowest quintile for the population at enrolment (calculated with principle components analysis using data on mother, household demographics and animal & other asset ownership) 2) Low parental education–no education or primary-schooling only (asked at enrolment) 3) Father occupation—father did not work, was seasonably employed or was a casual labourer at 12-month assessment 4) Mother married under the legal age of 18 years (reported at 12 month assessment) 5) Family debt—mother reported family debt or being unable to afford to buy food for herself or her child at any point between becoming pregnant and the 12 month assessment.

**Group 2—Maternal stress—Consists of six factors:** 1) Death of one or more of mother’s close family members since becoming pregnant reported at 12-month assessment 2) Mother seriously injured or ill 3) Any violence towards mother from husband (assessed using WHO multi-country study on women’s health and domestic violence against women[20]) or any other person since becoming pregnant reported at 12-month assessment 4) mother screens positive for mild, moderate or severe depression on PHQ9 or answers ‘yes’ to PHQ9 question on suicidal ideation (at 12-month assessment). PHQ9 is one of the most commonly used screening tools for depression and has been used widely in India[21] 5) Low level of support or high stress from others around the mother using the Duke social support & stress scale[22] reported at 12-month assessment 6) Problematic husband alcohol use reported by mother at 12-month assessment

**Group 3—Relationship—Consists of four factors:** 1) Any family member was unhappy when they found out that the child was a girl 2) Moderate or high concern level on Mother Object Relations Scale–short form (MORS-SF) at 12-month assessment. MORS-SF is a screening tool consisting of 14 short statements which a mother is asked to rate on a Likert-type scale to identify potential problems in early mother-infant relationship[23] 3) Very low quality interactions observed during a feeding episode at the 12-month assessment (assessed by non-specialist fieldworkers using the observed feeding index, a tool developed in this project where feeding is scored using tick-boxes. This tool will be published in due course). Very low quality means that the following was observed during the feeding episode: < = 1 positive talk by mother towards child, and < = 1 episodes of playful feeding and < = 1 responsive feeding actions, plus one or more negative actions such as force feeding, holds child’s head still to give food, shaking, threatening, shouting or berating observed by the mother towards child during feeding session 4) Lowest quintile score on HOME inventory measuring quality of the home environment through observations of the home and questions to the mother (total of 45 items, each scored 0 or 1) over the course of one hour [24] at 12 month assessment–the cut-off for the quintile fell between 27 & 28 points and the lowest of these (27 points) was chosen to create a conservative estimate of this factor.

**Group 4—Child—Consists of six factors:** 1) Child born prematurely (asked at 12-month assessment) 2) Child hospitalised in first year of life 3) Separation of mother & child for more than a week in the first year of life 4) Inadequate care–child left alone or with a child under 10 years for more than one hour in the past week (assessed at 12-month assessment) (From [25]) 5) Older children in the house say anything to make child cry or unhappy (in last week) (at 12-month assessment) (From [25]) 6) Older children who live in house: hit/punched/kicked/bit child on purpose to make them unhappy (in last week) (assessed at 12 month assessment)

A systematic cultural adaptation process based on Khan & Avan[26] was used. This comprised of six steps and aimed to ensure that each item was assessing the construct it was attempting to understand. Each item was first written in English, and the process of adaptation was: 1) Translation into Hindi independently by two trained research associates 2) Comparing these translations & assessing technical equivalence of these, then producing final translations by consensus for testing 3) Field research with project staff and mothers of young children to test understanding of translations and to improve them 4) Finalisation of tool for pretesting 5) Pretesting in the community to assess usability, 6) Assessor training, establishing inter-rater reliability and Pilot-testing.

For the 10 mothers with twins, questions relating to the child (e.g child hospitalisation) were asked for each child, and those relating to the mother herself (e.g maternal depression) were asked only once and answers applied to each child.

#### Growth & development measures

We trained assessors to do child development assessments at 18 months of age using the motor, cognitive & language scales of the Bayley Scales of Infant Development 3^rd^ Edition (BSID-III) in the home[27]. Assessors did two BSID-III assessments per day in pairs. Each assessment took 2–3 hours to complete. Each BSID-III scale consists of a series of progressively more difficult activities which children are asked to do whilst interacting with an assessor. Each item was scored 1 if the activity was demonstrated, otherwise it was scored 0. Assessment on each scale started at the item marked ‘K’ (start point for 16.5–19.5 month old children). Children not able to achieve three activities at that level were assessed as far as two levels back (the item marked ‘I’, which is the start point for 11–13.5 month old children) before the assessment was stopped. The assessment on each scale ended when the child scored 0 on five consecutive activities. We did comprehensive cultural adaptation and inter-rater reliability (IRR) checks, finding mean agreement between assessors of greater than 97%.

The same fieldworkers did anthropometrical measurements of children. Weight was measured to the nearest 0.01Kg using SECA-384 electronic scales which were calibrated weekly. Weighing was ideally done with the child’s clothes removed. If this was not possible, the child was weighed fully-clothed, then the clothes were removed and weighed. The difference between the weight of the fully-clothed child and the weight of the clothes was calculated to give the child’s weight. Length was measured to the nearest 0.1cm using the SECA-417 infantometer by two assessors as follows. The child was laid down on the infantometer board. The first assessor cupped their hands over the child’s ears and held the head against the end of the measurement board. The second assessor then ensured that the child’s body was straight on the board, placed one hand on the child’s legs to stabilise them and brought the footpiece upwards towards the child’s feet which were held perpendicular to the board. This assessor then read aloud the length board reading and this was recorded by the first assessor.

There were therefore three development outcomes & two growth outcomes assessed at 18 months of age.

### Sample size

One sample size calculation was done for the whole SPRING-ELS substudy, and the minimal sample size was exceeded in the work presented in this paper. A minimum sample size of 25 children per cluster was chosen for the overall SPRING-ELS substudy to give 90% power at the 5% level of significance to explore a range of adversities with prevalence of 20% to 80% and to detect effect sizes between 0.4SD & 0.5SD (assuming an intra-cluster correlation of 0.05) using an established formula[28].

### Data analysis

#### Adversities

Table 1 shows that five adversities had missing data. Four were missing less than 4%, and the fifth was missing 32.8%. We assumed these were missing-at-random and used multiple imputation by chained equations (MICE)[29], including all explanatory and outcome variables in each analysis. We used 30 imputations. We calculated descriptive data using a combination of all imputations.

We summed the 22 adversities described to create a total adversity score following a cumulative-adversity model [30–32] which recognises that children can be resilient to single adversities, but that combinations of these may be more harmful and overwhelm protective factors in a child’s life. In addition to this overall measure, we summed adversities within each of the four categories. This gave a total of five primary explanatory variables.

To ensure that summing adversities in this manner was not ‘double counting’ adversities (because children who had one adversity may be more likely to have several other related adversities) we used principle-components-analysis (PCA) to capture the linear combination of adversities which creates the maximum variance in the data, a similar manner to calculation of wealth indices[33]. We converted the raw PCA score into adversity quintiles giving five groups of children for analysis, giving a sixth explanatory variable.

#### Development measures

Raw scores for the BSID-III scales were converted to composite scores for each child following the BSID-III manual, based on the child’s age at assessment. This is done because BSID-III scores change quickly with age at this stage of development.

#### Growth measures

We converted child length and weight to height-for-age and weight-for-age z-scores using the zscore06 package for Stata15[34] which is based on the 2006 WHO child growth standards[35]. Using z-scores for these growth measures allowed child length & weight to be compared with international standards based on healthy breastfed children who on a population-level grow with the same distribution and trajectories wherever in the world they live.

### Association of cumulative adversity and growth & development outcomes

We used Stata 15 for all statistical analyses (StataCorp LLC: College Station, TX, USA). We used mixed-effects linear regression, accounting for trial cluster as a random effect and trial arm allocation as a fixed effect to calculate the adjusted mean growth and development values at each level of cumulative adversity and adversity quintile. This allowed us to examine the change in these outcomes as children were exposed to incrementally greater adversity. Because only 4% of children had a total adversity score of nine or more, we combined these with the children with a score of 8 to create an 8+ group for this analysis. Scatter diagrams suggested a linear relationship and we next we created models treating each of adversity score and adversity quintile as continuous variables to calculate the change in each of the five outcomes for a one-unit change in cumulative adversity.

### Association of adversity domains and growth & development outcomes

We used a similar model to explore the relationship between each of the four individual adversity domains and outcomes adjusted only for clustering and trial arm allocation. We then analysed the four domains together in a mutually adjusted multivariate model to examine the interrelationships between them with respect to outcomes.

### Ethics

Ethics approval was obtained from the London School of Hygiene & Tropical Medicine research ethics committee (SPRING: 23 June 2011, approval number 5983; SPRING-ELS substudy 19 May 2015, approval number 9886) and the Sangath Institutional Review board (IRB) (SPRING: 19 February 2014; SPRING-ELS substudy 27 May 2015). Approval was also granted by the Indian Council of Medical Research’s Health Ministry Screening Committee (HMSC) (SPRING: 24 November 2014; SPRING-ELS substudy: 6 October 2015). The SPRING trial is registered with clinicaltrials.gov, number NCT02059863. Informed written consent was obtained from mothers at enrolment into the trial surveillance system and again before a child’s first birthday for detailed developmental assessments.

### Role of funding source

The work was funded by the Wellcome Trust through two awards: a Wellcome Trust Research Training Fellowship to Sunil Bhopal (107818/Z/15/Z) & a Wellcome Trust Strategic Award for the SPRING Programme (0936115/Z/10/Z) for which Betty Kirkwood is the principle investigator. The funder had no role in the design and conduct of the study; collection, management, analysis, and interpretation of the data; and preparation, review, or approval of the manuscript. SB & BK have complete access to the study data and are responsible for the reported study findings, and made the decision to submit for publication.

## Results

### SPRING-ELS sample description

**Fig 2:**
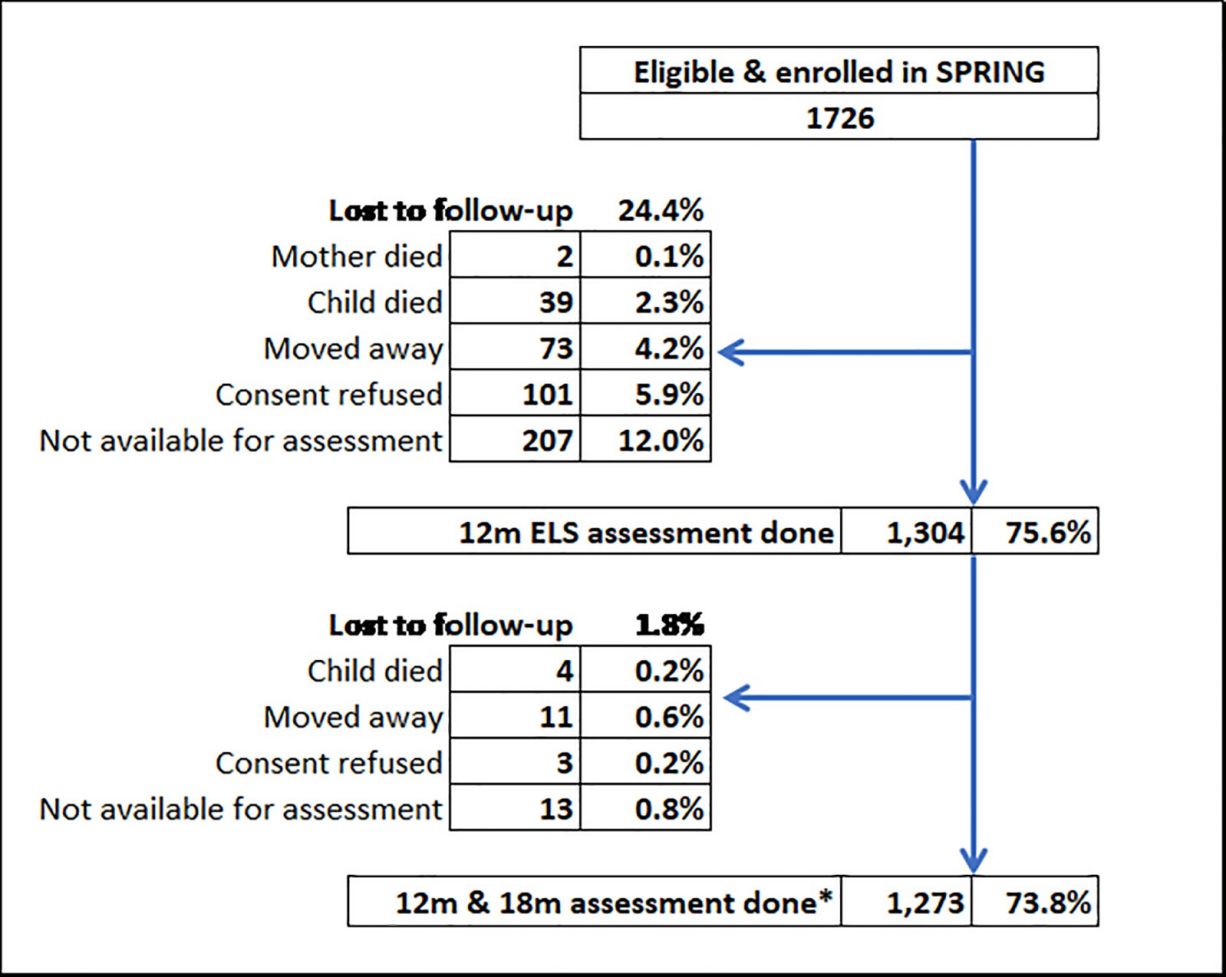
SPRING-ELS sub-study–flowchart of final assessment sub-sample *inclusion in this paper’s analysis sample requires both a 12 and 18 month assessment to be done.

SPRING enrolled 1726 eligible children soon after birth and aimed to assess all of those available at 12 and 18 months of age. 18 additional children were not eligible for enrolment because they were not living with their mother, had a congenital anomaly or because their mother was not capable of doing assessments. The flowchart in Fig 2 shows that we assessed 1273 (73.8%) children at both 12 and 18 months of age (between 6 July 2016 and 16 October 2017) in SPRING-ELS. This was a mean of 53 children per cluster (range 50–61).The majority of loss to follow-up was between enrolment & 12 months of age because families were not available for assessment (12.8%), refused consent (5.9%), had moved away (4.2%) or because of the death of the mother or child (2.6%).

Table 2 provides an overview of the demographic characteristics of the sample, and shows that there was no evidence of selection bias with regards to maternal education, caste, socioeconomic scores, sex, being a twin/triplet, and mother’s age at delivery in children lost to follow up compared to those in the assessment sample. There was a small difference in proportion of deliveries in a health facility, with a small p-value, however prevalence of facility-delivery was extremely high in both groups.

**Table 2:** Comparison of children completing ELS sub-study with those lost to follow up (* adjusted for clustering).

Indicator	Completed ELS assessment (C)	Lost to Follow Up (L)	C-L Difference * (95% CI)	p
Children in sample	1,273	453		
**% No maternal education (n)**	6.2% (79)	7.5% (34)	-1.25% (-3.96, 1.45)	0.364
**% scheduled/backward caste/tribe (n)**	60% (764)	60% (272)	-0.06% (-5.85, 5.73)	0.985
**% poorest (lowest 2 quintiles) (n)**	43% (548)	37.7% (171)	4.35% (-1.09, 9.80)	0.117
**% Male (n)**	53.4% (680)	55.4% (251)	-2.03% (-7.38, 3.32)	0.456
**% Twins/Triplets (n)**	1.6% (20)	1.1% (5)	0.26% (-0.56, 1.08)	0.538
**% Delivered in facility (n)**	98.2% (1250)	96% (435)	2.15% (0.22, 4.08)	0.029
**Mean age of mother at delivery (sd)**	22.3 (3.8)	22.3 (3.6)	0.031 (-0.374, 0.437)	0.879
**Mean SES score (sd)**	-0.15 (2.69)	0.02 (3.08)	-0.114 (-0.408, 0.180)	0.445

**Fig 3:**
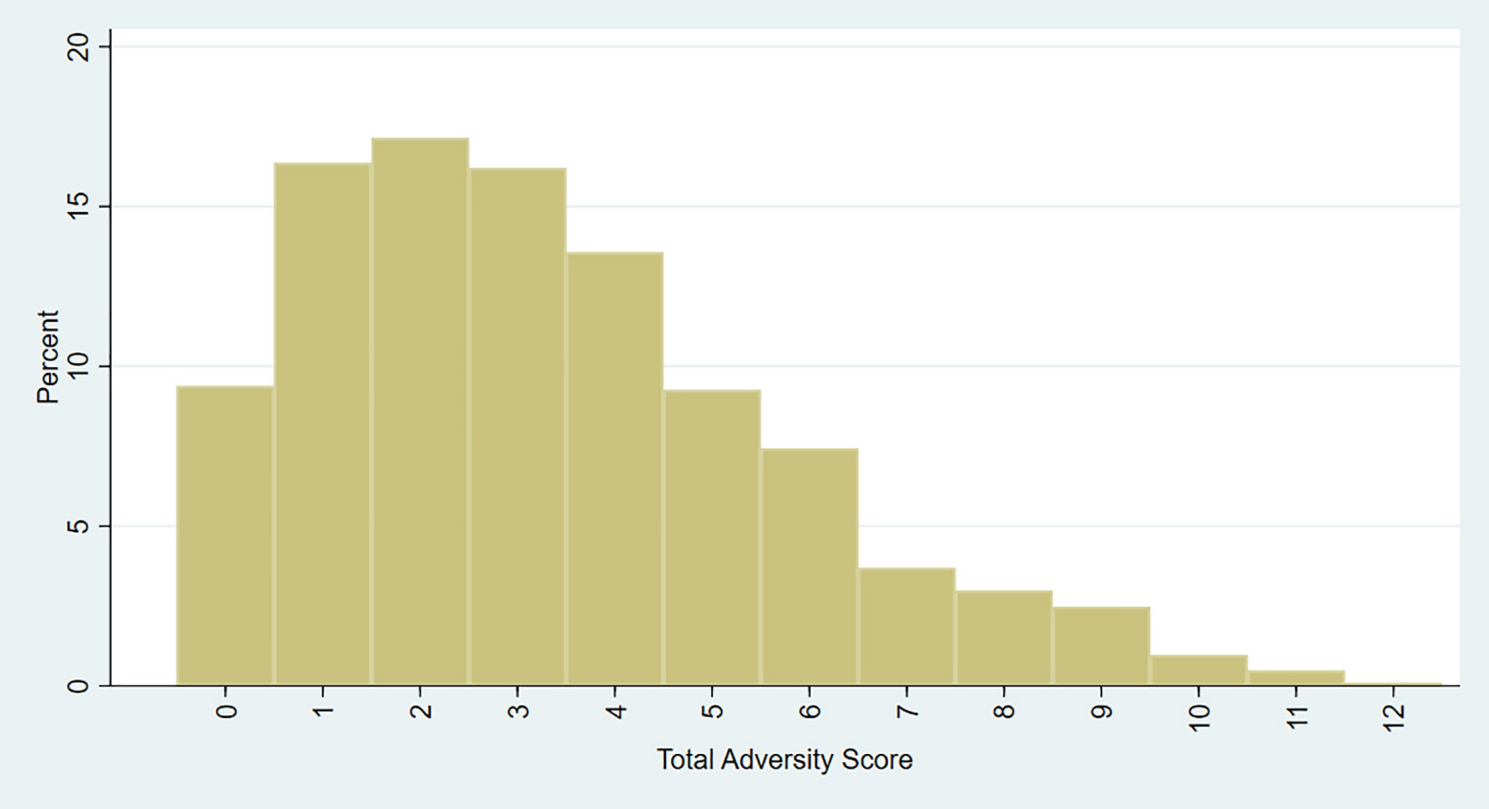
Proportion of children in the SPRING-ELS sub-study with each total adversity score.

The histogram in Fig 3 shows that 9.3% of children had no adversities, the proportion with one, two and three adversities was 16–17% for each, and that 42% of children had four or more adversities. The mean total adversity score was 3.3 (SD 2.4; range 0–12).

**Fig 4:**
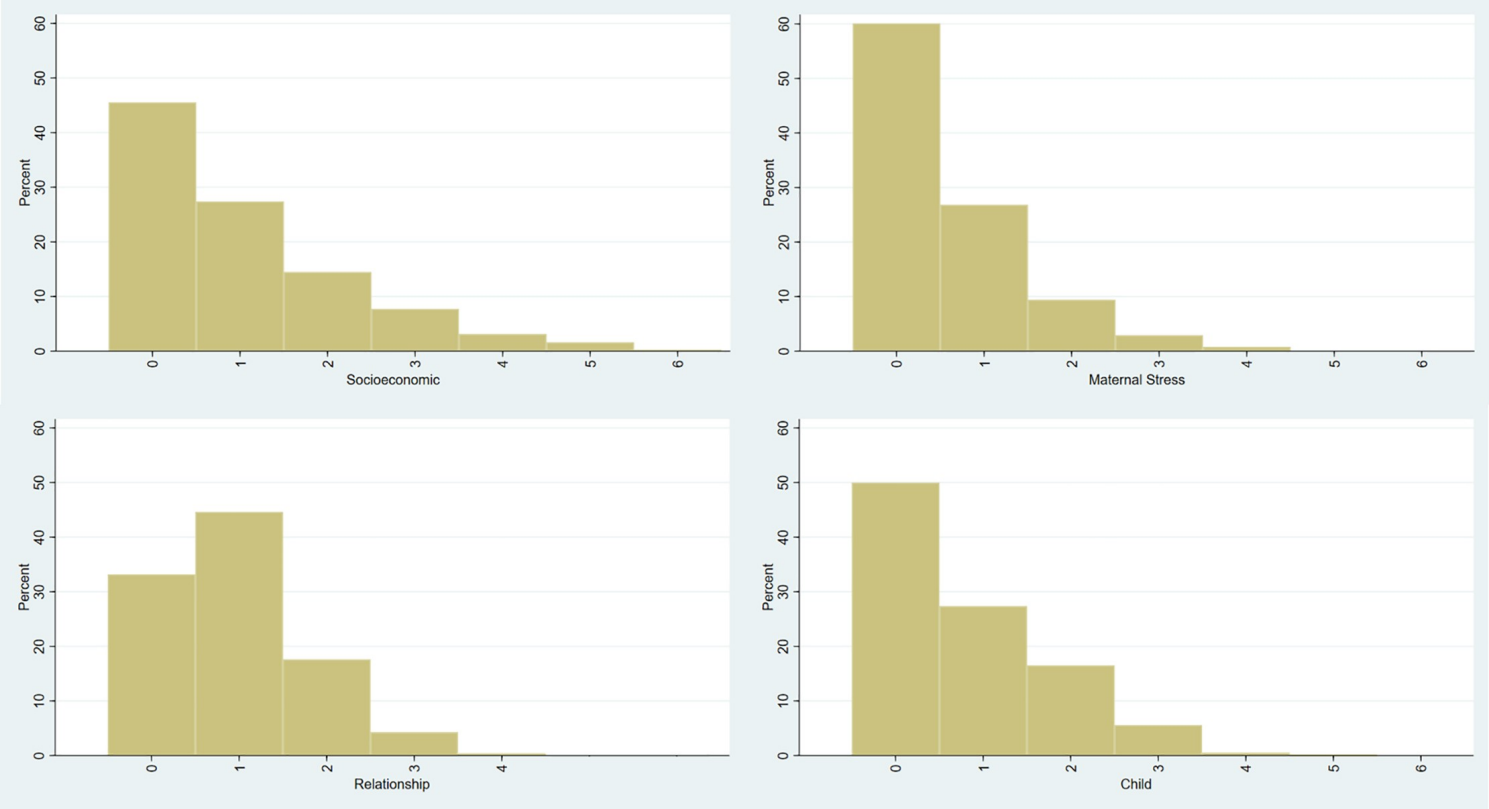
Histograms showing adversity scale scores for children in SPRING-ELS study for each of A) Socioeconomic B) Maternal Stress C) Relationship D) Child.

The four histograms in Fig 4 illustrate the distribution of adversity scale scores. More than half of children had a socioeconomic scale score of 1 or more, and the maximum observed score was 6. For maternal-stress, around a third of children had a score of 1 or more with a maximum observed score of 4 out of a possible 6. For the relationship scale, most children had a score of 0 or 1 with a maximum observed score of 3 out of a possible 4. For the child scale, half of children had a score of 0 and the maximum score observed was 5 out of a possible 6.

### Associations between cumulative adversity and growth & development

**Fig 5:**
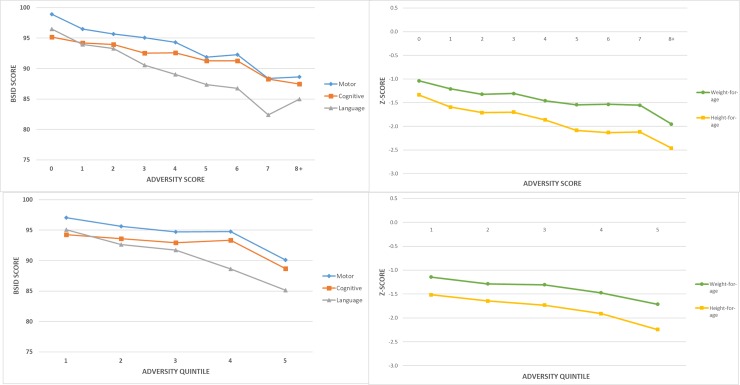
**Regression model associations between adversity and growth & development at 18 months of age in children enrolled in SPRING-ELS as follows:** A) Cumulative adversity & Development B) Cumulative adversity & Growth C) Adversity Quintile & Development D) Adversity Quintile & Growth.

There were consistent and strongly negative relationships between all measures of childhood adversity and all five child growth & development outcome measures at 18 months of age. The upper half of Table 3 shows the predicted mean development (left side) and growth (right side) outcomes at each total adversity score. Children with an adversity score of 0 had the highest scores in all of the motor, cognitive and language developmental domains, and were the least undernourished. These outcomes all worsened for each additional adversity as illustrated in Fig 5, Panels A & B with no evidence of a threshold effect. This figure also shows that the of the three developmental domains, language had the greatest slope of decrease for adversity.

**Table 3:** Association between childhood adversity and growth & development outcomes at 18 months of age.

Total Adversity Score	Number of children^1^	% ^1^	**Mean Bayley Scales of Infant Development III scores**^**2**^						**Mean Anthopometric Measures**^**2**^			
			Motor	95% CI	Cognitive	95% CI	Language	95% CI	Weight-for-age	95% CI	Height-for-age	95% CI
0	119	9.4%	98.9	(97.1, 100.7)	95.2	(93.0, 97.3)	96.5	(93.6, 99.4)	-1.04	(-1.22, -0.86)	-1.34	(-1.53, -1.14)
1	208	16.4%	96.5	(95.0, 97.9)	94.2	(92.5, 95.9)	93.9	(91.6, 96.3)	-1.21	(-1.35, -1.07)	-1.59	(-1.75, -1.44)
2	218	17.1%	95.7	(94.3, 97.1)	94.0	(92.3, 95.6)	93.3	(91.0, 95.5)	-1.32	(-1.46, -1.18)	-1.71	(-1.86, -1.56)
3	206	16.2%	95.1	(93.6, 96.6)	92.5	(90.8, 94.3)	90.6	(88.2, 92.9)	-1.31	(-1.45, -1.16)	-1.70	(-1.86, -1.55)
4	173	13.6%	94.3	(92.7, 95.9)	92.6	(90.8, 94.4)	89.0	(86.5, 91.5)	-1.46	(-1.62, -1.30)	-1.86	(-2.04, -1.69)
5	118	9.3%	91.9	(89.9, 93.8)	91.3	(89.1, 93.5)	87.4	(84.5, 90.3)	-1.54	(-1.74, -1.35)	-2.09	(-2.29, -1.88)
6	95	7.4%	92.3	(90.2, 94.4)	91.3	(89.0, 93.6)	86.8	(83.7, 89.9)	-1.54	(-1.75, -1.32)	-2.13	(-2.35, -1.91)
7	47	3.7%	88.4	(85.5, 91.3)	88.3	(85.1, 91.4)	82.4	(78.1, 86.6)	-1.56	(-1.86, -1.25)	-2.12	(-2.43, -1.81)
8+	89	7.0%	88.6	(86.6, 90.7)	87.4	(85.1, 89.7)	85.0	(81.9, 88.1)	-1.95	(-2.16, -1.74)	-2.46	(-2.68, -2.24)
Decrease per adversity (linear model)			-1.1	(-1.3, -0.9)	-0.8	(-1.0, -0.6)	-1.4	(-1.8, -1.1)	-0.09	(-0.11, -0.06)	-0.12	(-0.14, -0.09)
p-trend			<0.001		<0.001		<0.001		<0.001		<0.001	
Adversity quintile	1		97.1	(95.8, 98.4)	94.3	(92.7, 95.8)	95.1	(92.9, 97.2)	-1.14	(-1.27, -1.01)	-1.52	(-1.66, -1.38)
	2		95.6	(94.3, 97.0)	93.6	(92.1, 95.2)	92.6	(90.5, 94.8)	-1.29	(-1.42, -1.15)	-1.64	(-1.78, -1.50)
	3		94.7	(93.4, 96.0)	92.9	(91.4, 94.4)	91.7	(89.6, 93.9)	-1.30	(-1.43, -1.18)	-1.73	(-1.87, -1.59)
	4		94.8	(93.5, 96.1)	93.4	(91.9, 94.9)	88.6	(86.5, 90.8)	-1.47	(-1.60, -1.34)	-1.91	(-2.05, -1.77)
	5		90.1	(88.8, 91.4)	88.7	(87.2, 90.2)	85.2	(83.0, 87.3)	-1.71	(-1.84, -1.58)	-2.24	(-2.38, -2.10)
Decrease per quintile (linear model)			-1.5	(-1.9, -1.1)	-1.1	(-1.5, -0.7)	-2.4	(-2.9, -1.8)	-0.13	(-0.17, -0.09)	-0.17	(-0.21, -0.13)
p-trend			<0.001		<0.001		<0.001		<0.001		<0.001	

Note: Total adversity score represents the summed score of 22 possible adversities. Adversity quintiles are based on principle components analysis (1 represents the least adverse group, 5 the most).

^1^ because of multiple imputation, numbers & percentage of children in each total adversity score group is an estimate based on combinations of the imputed datasets

^2^estimated mean value at each adversity level, using multiple-imputation

Results treating total adversity score as continuous in the regression model are presented in the grey bar at the bottom of the upper half of the Table 3. For motor this was a 1.1 point decrease per adversity (95%CI -1.3, -0.9); for cognitive 0.8 points (95%CI -1.0, -0.6); for language 1.4 points (95%CI -1.9, -1.1). For growth this was -0.09 change in weight-for-age z-score (-0.11, -0.06) and -0.12 for height-for-age (-0.14, -0.09).

The bottom half of Table 3 shows similar strong and negative relationships using adversity quintiles, albeit with narrower differences between most and least adversity. The decrease from the 4^th^ to 5^th^ (most disadvantaged) quintiles is greater than for other quintile changes.

### Associations between domains of adversity and growth & development

**Fig 6:**
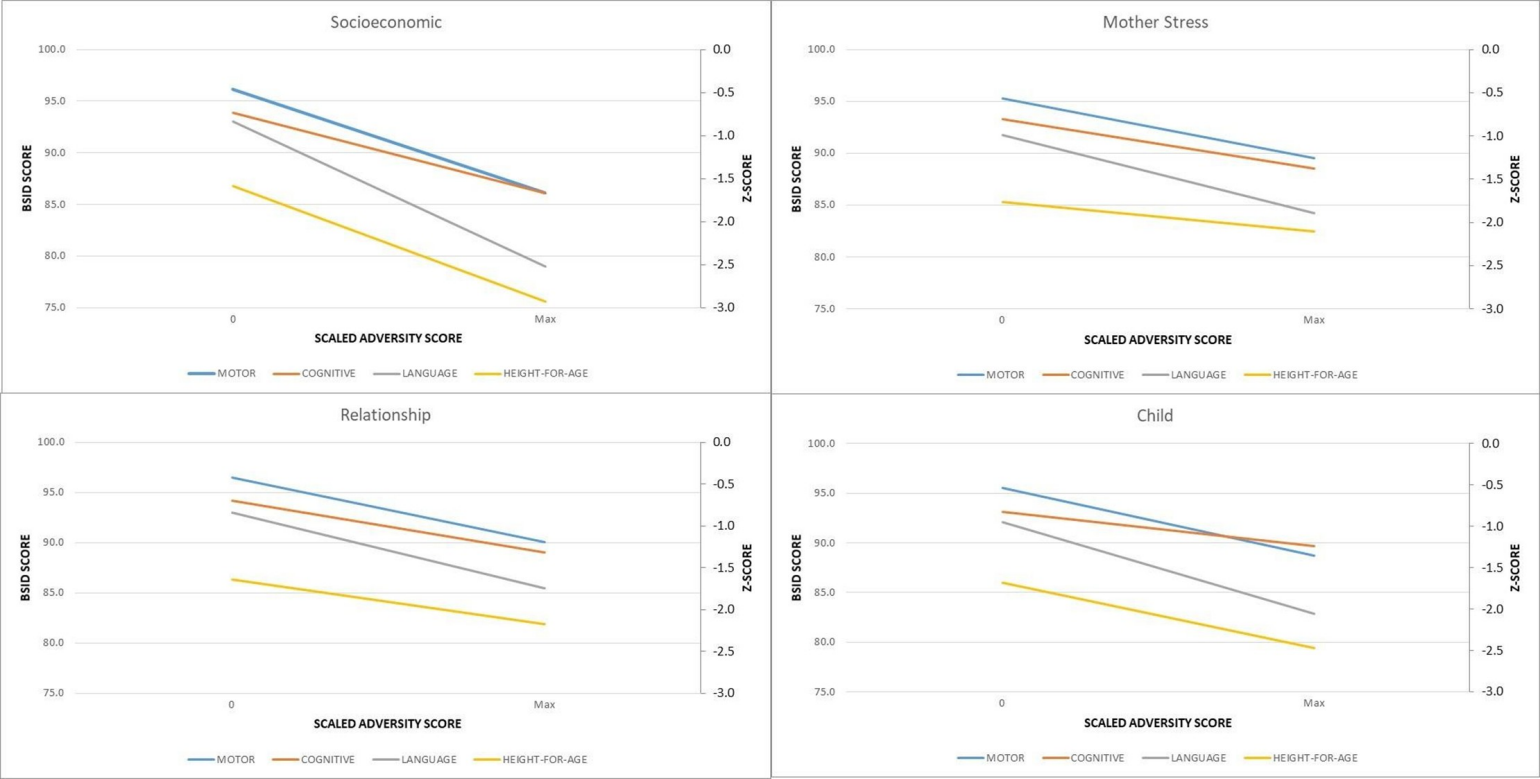
**Regression model associations between adversity scales and growth & development at 18 months of age in children enrolled in SPRING-ELS as follows:** A) Socioeconomic Score B) Maternal Stress C) Relationship D) Child. Note: weight-for-age described in text but not shown to aid clarity of figure.

Table 4 shows the strong and consistent negative relationships between adversity domains and outcomes. For each regression model the mean growth or development outcome at an adversity scale score of 0 is presented, along with a change with each increase in adversity using a linear model. These relationships are illustrated in Fig 6 showing the adversity score (x-axis) scaled between 0 adversities and the maximum score observed.

**Table 4:** Association between four adversity scales and growth & development outcomes at 18 months of age.

Adversity scale	Items	Max observed		Bayley Scales of Infant Development			Anthropometry	
				Motor	Cognitive	Language	Weight-for-age	Height-for-age
Socioeconomic	6	6	Mean at 0 (95% CI)	96.2 (95.3, 97.1)	93.9 (92.8, 95.0)	93.0 (91.3, 94.7)	-1.20 (-1.28, -1.13)	-1.58 (-1.67, -1.49)
			Change with increase (95% CI)	-1.7 (-2.1, -1.2)	-1.3 (-1.8, -0.8)	-2.3 (-3.0, -1.7)	-0.18 (-0.22, -0.13)	-0.22 (-0.27, -0.18)
			p for slope	<0.001	<0.001	<0.001	<0.001	<0.001
Maternal stress	6	4	Mean at 0 (95% CI)	95.3 (94.4, 96.2)	93.3 (92.2, 94.3)	91.7 (90.0, 93.4)	-1.33 (-1.41, -1.24)	-1.76 (-1.86, -1.66)
			Change with increase (95% CI)	-1.4 (-2.1, -0.8)	-1.2 (-1.9, -0.5)	-1.9 (-2.8, -1.0)	-0.10 (-0.16, -0.03)	-0.09 (-0.16, -0.02)
			p for slope	<0.001	0.001	<0.001	0.004	0.017
Relationship	4	3	Mean at 0 (95% CI)	96.5 (95.4, 97.5)	94.2 (93.0, 95.4)	93.0 (91.1, 94.9)	-1.25 (-1.35, -1.16)	-1.64 (-1.75, -1.53)
			Change with increase (95% CI)	-2.1 (-2.8, -1.5)	-1.7 (-2.4, -1.0)	-2.5 (-3.4, -1.6)	-0.14 (-0.20, -0.07)	-0.18 (-0.25, -0.10)
			p for slope	<0.001	<0.001	<0.001	<0.001	<0.001
Child	6	5	Mean at 0 (95% CI)	95.6 (94.6, 96.5)	93.1 (92.0, 94.2)	92.1 (90.3, 93.9)	-1.32 (-1.40, -1.23)	-1.69 (-1.79, -1.58)
			Change with increase (95% CI)	-1.4 (-1.9, -0.8)	-0.7 (-1.3, -0.1)	-1.9 (-2.6, -1.1)	-0.08 (-0.14, -0.03)	-0.16 (-0.22, -0.10)
			p for slope	<0.001	0.026	<0.001	<0.001	<0.001

Table 5 displays the results of five mutually adjusted analyses examining the interrelationship between adversity scales and each outcome. Here all of the relationships in Table 4 are somewhat attenuated, but the majority still show strong negative associations. The exception is the mother-stress scale, which has smaller point estimates and confidence intervals that cross zero indicating that this scale is less associated with outcomes. In addition, the association between adversity and weight-for-age appears to be mostly accounted for by the socioeconomic scale as the point estimates for the remaining three scales reduce considerably.

**Table 5:** Results from five regression models assessing the combined effect of all four adversity subscales on each growth & development outcome at 18 months of age.

Adversity scale	Items	Max observed		Bayley Scales of Infant Development			Anthropometry	
				Motor	Cognitive	Language	Weight-for-age	Height-for-age
Socioeconomic	6	6	Change with increase (95% CI)	-1.3 (-1.8, -0.9)	-1.0 (-1.5, -0.5)	-1.9 (-2.6, -1.3)	-0.16 (-0.21, -0.11)	-0.21 (-0.26, -0.16)
			p for slope	<0.001	<0.001	<0.001	<0.001	<0.001
Maternal stress	6	4	Change with increase (95% CI)	-0.4 (-1.1, 0.2)	-0.4 (-1.2, 0.3)	-0.6 (-1.5, 0.4)	-0.01 (-0.08, 0.06)	0.04 (-0.03, 0.11)
			p for slope	0.178	0.232	0.241	0.840	0.264
Relationship	4	3	Change with increase (95% CI)	-1.5 (-2.2, -0.9)	-1.3 (-2.1, -0.6)	0.5 (-2.6, -0.7)	-0.08 (-0.14, -0.01)	-0.11 (-0.18, -0.04)
			p for slope	<0.001	0.001	<0.001	0.031	0.003
Child	6	5	Change with increase (95% CI)	-0.9 (-1.5, -0.4)	-0.3 (-0.9, 0.3)	0.4 (-2.1, -0.5)	-0.05 (-0.11, 0.01)	-0.12 (-0.18, -0.06)
			p for slope	0.001	0.321	0.001	0.087	<0.001
Mean when all adversity scales = 0				98.3 (97.2, 99.3)	95.4 (94.0, 96.7)	95.5 (93.6, 97.4)	-1.11 (-1.21, -1.00)	-1.42 (-1.53, -1.31)
p for overall model fit				<0.001	<0.001	<0.001	<0.001	<0.001

## Discussion

We did a population-based study in rural India and followed up mothers and their infants through pregnancy and the first 18 months of life. We found that most children faced one or more adversity and nearly 50% faced four or more of these potential impediments to wellbeing. The key finding was that each extra increase in childhood adversity was associated with both poorer growth and also poorer development measured at 18 months, a crucial time for optimal brain development and a key predictor of future health and wellbeing. This finding was described both using the overall cumulative adversity measure of 22 adversities, and four subscale measures each made up of 4–6 items. There was no evidence of a threshold effect in either the growth or development models, with each additional adversity being associated with progressively poorer outcomes. This adds to the case for a cumulative-risk approach to adversity, supporting the notion that it is the accumulation of multiple factors that is detrimental to child wellbeing in this context rather than specific adversities. Finding similar results using the adversity quintiles derived from the PCA analysis corroborates this finding, suggesting that individual factors contribute independently to the associations with impaired outcomes. When the adversity scales were added into individual regression models assessing the combined effects of the scales on each outcome, the point estimates for each were somewhat attenuated, suggesting that there was some overlap between the scales. Notably, in these models, the maternal stress scale point estimates were considerably attenuated with 95% CI crossing zero suggesting that the other scales accounted for much of the association seen in the initial analysis. This is an unexpected finding as these sorts of adversities–including maternal stress and depression—are some of the first to be considered when considering infant wellbeing.

Socioeconomic adversities were strongly associated with impaired growth & development, and are not addressed directly through SPRING. There is, however, evidence to support prioritisation of socioeconomic improvement for families because of benefits to the youngest members of society–for example, the cash-transfer schemes in Mexico’s Opportunidates[36] and Nigaragua’s Atención a Crisis[37] and our results suggest that other programmes may wish to explore this further, particularly given that early childhood programs may exacerbate existing developmental inequalities (e.g described by Victora et al in Brazil[38]) if uptake of promoted activities is greater in higher socio-economic groups with already comparatively better growth & development.

Some of the other adversities, including carer-child relationship are more clearly modifiable through SPRING’s home-visiting approach, and it is therefore crucial that programs examine the extent to which interventions address a broad range of adversities, including those that are culturally specific and important–for example, in this study context, family desire for a boy-child was prevalent.

Growth data give a clear reminder of the appalling anthropometric status of children in this area. The mean height-for-age z-score and 42.2% prevalence of stunting are amongst some of the worst reported worldwide. Of note, even those children living with the least adversity have growth far below the expected norms for age (weight for age -1.04SD (95% CI -1.22, -0.86) below the mean; height for age -1.34SD (95% CI -1.53, -1.14) below the mean—this was much worse for those children with higher adversity scores. Addressing adversity is likely to go some way towards improving growth status of children, but we do not provide evidence here that suggests that this alone would bring this up to global norms. The differences seen in development between those with the least (zero adversity) and the most (8+ adversities) was considerable at more than 10 points on the BSID-III motor scale, nearly 8 points on the cognitive scale, and more than 11 points in the language scale. These differences are notable at the individual level and mean that these individual children are at risk of continuing suboptimal development through childhood.

Our findings are in accordance with the limited literature on young child growth & development. The study which comes closest to addressing the questions we set out to answer in similarly young infants is from Bangladesh[39] where Hamadani et al focussed on socioeconomic status and home stimulation as measures of adversity and examined the association with the mental developmental index of an earlier version of BSID. Other work tends to focus on individual adversities, particularly socioeconomic status, maternal depression & the home-environment (measured using the HOME inventory) and one or two domains of child development. For example, in the early 1980s, Agarwal et al reported associations between socio-economic status, family size & the HOME-inventory with developmental scores in 1–3 year olds in India[40]. Paxton & Schady reported poorer cognitive development in children in Ecuador by socioeconomic status[41]. Patel et al report poorer mental and motor development in 43 children of depressed mothers compared with controls in India[42]. Similarly, Galler reported poorer maternal mood was associated with poorer motor development in a sample of 92 infants in Barbados[43] and there is a report of poorer living conditions being associated with poorer Peabody scores[44]. In contrast to our approach, several studies treat adversities as possible confounders in the relationship between a particular adversity of interest and outcomes to delineate the contribution of a specific adversity. An example is a study that examined the relationship between common mental disorder scores (using the SRQ-20) & child growth/development in four LMICs. This is based on a conceptual framework that treats child, maternal & household characteristics as potential confounders and then statistically adjusts for these to examine adjusted risk ratios for the association[45]. We prefer the approach of considering many adversities around a child to understand their cumulative impact as described by Evans et al [46] in their review of different models of risk for child development, and by Wachs & Rahman[9] based on the understanding that children are likely to be able to manage individual adversities, but that the combination of these simultaneously is more highly detrimental to coping mechanisms. One limitation of this approach is that it is not possible to examine possible interactions between adversities–this is important because the impact of any one adversity is likely to be influenced by the presence or absence of other adversity and protective factors.

Ours is the first large population-based study in a LMIC to examine growth & developmental disadvantage in multiple domains faced by young infants living with a wide range of adversities relating to their own experiences, stressful experiences for their mothers, difficulties with carer-child interaction, and broader socioeconomic position of their household. Unlike previous studies, we assessed adversity prospectively from birth by identifying all pregnancies & births in our large study area through a trial surveillance system. We found good representativeness of those in our cohort compared with those identified by the trial surveillance system but not assessed. Other strengths include the robust approach we took to deal with a small amount of missing data, the use of a secondary principle components analysis to confirm the primary analysis based on total number of adversity factors, and the broad range of adversity factors analysed. We also used BSID-III–the gold-standard measurement tool which has been used worldwide including in India—to measure child development. We comment on between-group developmental differences in our study and not on absolute values because of complexities in cross-setting interpretation.

Given the nature of the analyses presented there is a possibility that unmeasured confounding, bias or common cause of both adversity and outcome—for example genetic differences and other characteristics at birth–account for some or all of the relationship described. Other study designs would be required to examine this further.

Our results are the first from a large population-based study in an LMIC to show that increasing adversity in multiple domains increases risks to child growth & development at a very early age. There is an urgent need to act to improve these outcomes for young children in LMICs and these findings suggest that Early Childhood programmes should prioritise early childhood adversity because of its impact on developmental inequities from the very start.
